# Multiple myeloma patients with a long remission after autologous hematopoietic stem cell transplantation

**DOI:** 10.1038/s41408-024-01062-2

**Published:** 2024-05-17

**Authors:** Oren Pasvolsky, Zhongya Wang, Denái R. Milton, Mark R. Tanner, Qaiser Bashir, Samer Srour, Neeraj Saini, Paul Lin, Jeremy Ramdial, Yago Nieto, Guilin Tang, Partow Kebriaei, Yosra Aljawai, Hina N. Khan, Hans C. Lee, Christine Ye, Krina K. Patel, Sheeba K. Thomas, Robert Z. Orlowski, Elizabeth J. Shpall, Richard E. Champlin, Muzaffar H. Qazilbash

**Affiliations:** 1 Department of Lymphoma and Myeloma, The University of Texas MD Anderson Cancer Center, Houston, TX USA; 2 Department of Biostatistics, The University of Texas MD Anderson Cancer Center, Houston, TX USA; 3 Department of Stem Cell Transplantation and Cellular Therapy, The University of Texas MD Anderson Cancer Center, Houston, TX USA; 4 Department of Hematopathology, The University of Texas MD Anderson Cancer Center, Houston, TX USA; 5 Department of Hematology/Oncology, McGovern Medical School, The University of Texas, Health Sciences Center at Houston, Houston, TX USA

**Keywords:** Myeloma, Prognosis

## Abstract

Autologous stem cell transplantation (autoHCT) is considered standard of care for newly diagnosed multiple myeloma (MM). Although most patients eventually progress after autoHCT, a small proportion achieve a durable response. In this retrospective study we included 1576 patients, 244 (15%) of whom were long-term responders (LTR), defined as having a progression-free survival (PFS) of ≥8 years after transplant. Patients in the LTR group were younger than the non-LTR group (median age 58.4 vs. 59.5 years; *p* = 0.012), less likely to have high-risk cytogenetics (4% vs. 14%; *p* < 0.001), more often had <50% bone marrow plasma cells (67% vs. 58%; *p* = 0.018) and R-ISS stage I disease (43% vs. 34%). More patients in the LTR group received post-transplant maintenance (63% vs. 52%; *p* = 0.002). Patients in the LTR group had higher rates of complete response (CR) at day100 (41% vs. 27%; *p* < 0.001) and at best post-transplant response (70% vs. 37%; *p* < 0.001), compared to the non-LTR group. Patients in the LTR groups had a median PFS of 169.3 months and the median overall survival (OS) had not been reached. The leading cause of death in the LTR was disease progression. In conclusion, 15% of patients in the cohort were LTR after upfront autoHCT, with distinct characteristics and a median PFS of more than 14 years.

## Introduction

Advancements in anti-myeloma therapeutics have led to improved outcomes for patients with multiple myeloma (MM). Use of proteosome inhibitors, immunomodulatory drugs (IMiD) and autologous stem cell transplantation (autoHCT) have extended the survival of MM patients by several years, and are considered standard of care for patients with newly-diagnosed MM (NDMM) [1]. However, most patients eventually progress [2, 3], and MM is generally considered an incurable disease. Interestingly, a small proportion of patients do achieve durable remission after autoHCT. Terpos et al. described a cohort of 406 MM patients treated at a single center in Greece between 1994 and 2010, and found that 9% (*n* = 36) of newly diagnosed MM patients experienced a progression-free survival (PFS) of at least 7 years [4]. Only 29% of patients in that cohort underwent upfront autoHCT.

Few reports have focused on MM patients with prolonged remissions after autoHCT; most of which had small numbers of patients and used varying methodologies. A single-center analysis from Spain identified 54 (22%) of 250 patients who were transplanted between 1990 and 2015 as patients with prolonged remission following autoHCT, defined as achieving a sustained response for more than 5 years after autoHCT [5]. A report from the Mayo Clinic identified 46 (9%) of 509 patients as exceptional responders after autoHCT, defined as having a PFS of at least eight years without any maintenance therapy [6].

With the advent of newer treatments, such as chimeric antigen receptor-directed T cells (CAR-T) and bispecific antibodies, it is important to identify patients that benefit the most from autoHCT. Therefore, in this study we sought to identify characteristics of patients who achieved a long remission after upfront autoHCT, and to evaluate the predictors of a long remission.

## Materials and methods

### Study design and participants

We conducted a retrospective, single-center, chart review study of patients with NDMM who received autoHCT between 2000 and 2014 at our center. Data was obtained from our institution’s transplant database and chart-based review. Long-term responders (LTR) were defined as patients who had a PFS of at least eight years following autoHCT, with or without post-autoHCT maintenance. The primary endpoints were PFS and overall survival (OS), and the secondary endpoints were hematological response and minimal residual disease (MRD) status after autoHCT. This study was conducted after approval by the institutional review board (IRB) at the University of Texas MD Anderson Cancer Center and was conducted in accordance with the Declaration of Helsinki and the 1996 Health Insurance Portability and Accountability Act (HIPAA).

### Response definitions and MRD evaluation

We used the International Myeloma Working Group (IMWG) criteria to evaluate the response and progression [7]. Patients were categorized as having complete response (CR), stringent CR (sCR), very good partial response (VGPR), partial response (PR), stable disease (SD), or progressive disease (PD).

MRD status was assessed using eight-color next-generation flow cytometry (NGF) with a sensitivity of 1/10^-5^ cells (0.001%), based on acquisition and analysis of at least two million events.

### Fluorescence in situ hybridization analysis

High-risk cytogenetic abnormalities were identified using fluorescence in situ hybridization (FISH) analysis. The following FISH probe sets were used for (4;14), t(14;16), del[17p], and 1q21 gain or amplification: IGH::FGFR3 dual-color dual-fusion probes; IGH::MAF dual-color dual-fusion probes; TP53/CEP17 dual-color and CDKN2C/CKS1B dual-color probes. The following cut-off values for common abnormal signal patterns were established by our clinical cytogenetics laboratory: 7.9% for 1q21 gain, 0% for 1q21 amplification, 4.7% for deletion of TP53, 0.4% for t(4;14), and 0% for t(14;16).

### Statistical methods

Demographics and clinical characteristics were summarized for all patients and by LTR status. Continuous variables were summarized using medians and ranges for patients with non-missing data and evaluated by Wilcoxon rank-sum test, while categorical variables were summarized using frequencies and percentages and assessed by Fisher’s exact test or its generalization.

PFS time was computed from autoHCT date to the date of progressive disease or death, whichever occured first; patients who remained alive without progressive disease were censored at their last follow-up time. OS time was computed from autoHCT date to the date of death or last follow-up. Patients who were still alive at their last follow-up date were censored at that time for OS.

PFS and OS were estimated using the Kaplan–Meier method, and the difference in survival curves between groups was assessed using log-rank test. Additionally, association between OS or PFS and measures of interest were evaluated using Cox proportional hazards regression models. Measures occurring after autoHCT, including hematologic response at day 100 and at best response, best MRD status post-transplant and maintenance therapy, were treated as time-dependent covariates in the models. In the multivariable analysis, all variables with *p*-values less than 0.1 in the univariate analysis were included in the final model. P-values less than 0.05 were considered significant in the multivariable analysis. All the statistical analyses were performed using SAS enterprise guide 7.15 HF7. No adjustments for multiple testing were included.

## Results

### Patient, disease, and treatment characteristics

Our analysis included a total of 1576 NDMM patients who underwent autoHCT at our institution, and 224 (14%) were identified as LTR. Patients in the LTR group were somewhat younger than the non-LTR group (median age 58.4 vs. 59.5 years; *p* = 0.012), and were less likely to have high-risk cytogenetic abnormalities (4% vs. 14%; *p* < 0.001). Patients in the LTR group more often had R-ISS stage I disease (43% vs. 34%) and less often had R-ISS stage III disease (3% vs. 10%) compared those in the non-LTR group (*p* = 0.010). Furthermore, patients in the LTR group more often had a low burden of disease in the bone marrow at diagnosis, defined as bone marrow plasma cells <50% (67% vs. 58%; *p* = 0.018). The most commonly used induction regimens in the entire cohort were IMiD+dexamethasone (30%) and bortezomib+lenalidomide+dexamethasone (VRD) (16%), without a significant difference in the type of induction regimen used between the two groups (*p* = 0.26). More patients in the LTR group received post-transplant maintenance therapy compared to those in the non-LTR group (63% vs. 52%; *p* = 0.002) (Table 1).

**Table 1:** Patient characteristics.

Measure	LTR	*p*-value^a^	
	Yes (*N* = 244)	No (*N* = 1332)	
Age at autoHCT (years)			0.012^b^
Median (range)	58.4 (31.7–79.4)	59.5 (29.4–80.6)	
Gender, *n* (%)			0.08
Male	130 (53)	792 (59)	
Female	114 (47)	540 (41)	
Race, *n* (%)			0.58
Black	44 (18)	222 (17)	
Non-Black	195 (82)	1087 (83)	
Light chain type, *n* (%)			0.11
Kappa	164 (68)	853 (64)	
Lambda	73 (30)	463 (35)	
Biclonal	4 (2)	9 (1)	
Cytogenetic risk, *n* (%)			<0.001
High^c^	9 (4)	168 (14)	
Standard	212 (96)	992 (86)	
Bone marrow plasma cell burden, *n* (%)			0.018
<50%	151 (67)	716 (58)	
≥50%	75 (33)	510 (42)	
R-ISS, *n* (%)			0.010
I	57 (43)	200 (34)	
II	72 (54)	332 (56)	
III	4 (3)	58 (10)	
HCT-CI score, *n* (%)			0.93
≤3	196 (80)	1072 (81)	
>3	48 (20)	258 (19)	
LDH, *n* (%)			0.19
Normal	133 (90)	624 (85)	
>ULN	15 (10)	106 (15)	
Creatinine, *n* (%)			0.37
≤2	194 (87)	994 (84)	
>2	30 (13)	188 (16)	
${{\boldsymbol{\beta }}}_{{\boldsymbol{2}}}$ microglobulin			0.043^b^
Median (range)	3.1 (1.1–34.1)	3.5 (0.5–81.4)	
Bone lesions, *n* (%)			0.49
0	66 (28)	312 (24)	
1–3	93 (39)	520 (40)	
>3	80 (33)	466 (36)	
Induction regimen, *n* (%)			0.26^d^
Chemotherapy	15 (6)	134 (10)	
ImiD+Dexa	70 (29)	401 (30)	
VCD	30 (12)	127 (10)	
Vd	31 (13)	203 (15)	
VRD	44 (18)	201 (15)	
VTD	20 (8)	105 (8)	
Other	34 (14)	161 (12)	
Hematologic response prior to autoHCT, *n* (%)			0.057
CR/sCR	20 (8)	106 (8)	
VGPR	103 (42)	444 (33)	
PR	106 (43)	707 (53)	
SD	15 (6)	72 (5)	
PD	0	3 (<1)	
Any post-transplant maintenance, *n* (%)			0.002
No	91 (37)	639 (48)	
Yes	153 (63)	693 (52)	

*autoHCT* autologous hematopoietic stem cell transplant, *CR* complete response, *Dexa* dexamethasone, *HCT-CI* hematopoietic cell transplantation comorbidity index, *ImiD* immunomodulatory drug, *LDH* lactate dehydrogenase, *LTR* long-term responders, *n* number, *PD* progressive disease, *PR* partial response, *R-ISS* Revised International Staging System, *sCR* stringent complete response, *SD* stable disease, *VCD* bortezomib, cyclophosphamide, dexamethasone, *Vd* bortezomib, dexamethasone, *VGPR* very good partial response, *VRD* bortezomib, lenalidomide, dexamethasone, *VTD* bortezomib, thalidomide, dexamethasone.

^a^Fisher’s exact test or generalization.

^b^Wilcoxon rank-sum test.

^c^Defined as t(4;14), t(14;16), del(17p) and 1q21 gain or amplification by fluorescence in situ hybridization.

^d^Chi-squared test.

### Responses and MRD outcomes

Patients in the LTR group more often achieved a hematological response of ≥VGPR prior to autoHCT (50% vs. 41%; *p* = 0.009), and more often had MRD negative status prior to autoHCT (52% vs. 35%; *p* = 0.016) compared to those in the non-LTR group. Of note, pre-transplant MRD status was missing in the majority of patients, 73% of the patients in the LTR group and 77% of the non-LTR group.

**Fig. 1:**
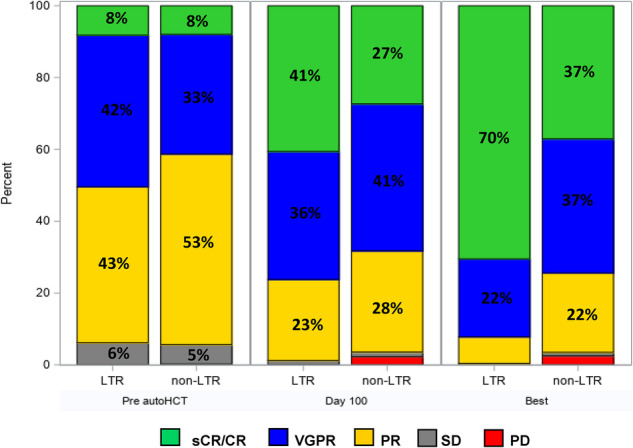
Hematological responses for long-term responders (LTR) and non-LTR: prior to transplant, at day 100 after transplant and at best post-transplant response.

At day 100 post-transplant, a higher percentage of patients in the LTR group had a hematological response of ≥ CR compared to those in the non-LTR group (41% vs. 27%; *p* < 0.001). Similarly, at best post-transplant response evaluation, a higher percentage of patients in the LTR group had a hematological response of ≥CR compared to the non-LTR group (70% vs. 37%; *p* < 0.001). Hematological responses prior to autoHCT and following autoHCT in the LTR and non-LTR groups are presented in Fig. 1.

### Survival outcomes

The median follow-up time for the entire cohort was 83.7 (range 0.2–262.0) months. As expected, patients in the LTR group had longer median follow-up compared to the non-LTR group [126.1 (range 96.0–254.9) months vs. 73.9 (range 0.2–262.0) months; *p* < 0.001]. Median follow-up among survivors was 127.3 (range 96.0–254.9) months in the LTR group and 99.3 (range 0.9–262.0) months in the non-LTR group (*p* < 0.001).

**Fig. 2:**
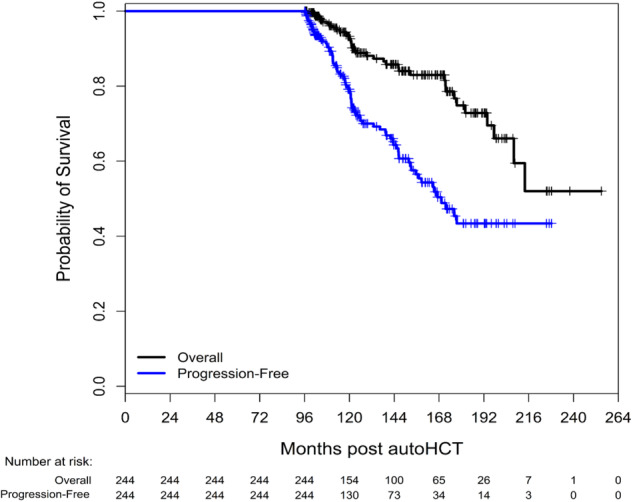
Progression-free survival (blue curve) and overall survival (black curve) for long-term responders.

Median PFS in the LTR and non-LTR groups was 169.3 months and 26.5 months, respectively. Median OS was not reached (208.1 months-not reached) in the LTR group and 81.3 months (77.5–85.7) in the non-LTR group. Kaplan–Meier survival curves for the LTR group are presented in Fig. 2. Time-to-progression curve for the LTR group is shown in Supplementary Fig. 1. The most common cause of death among the LTR was progressive MM (35%), followed by second primary malignancies (22%) (Supplementary Table 1). In the non-LTR group, 159 (12%) patients developed a second primary malignancy, compared to 36 (15%) in the LTR group (*p* = 0.24).

In multivariable analysis for PFS for all patients (Table 2), achieving pre-transplant MRD negative ≥VGPR [hazard ratio (95% CI) 0.64 (0.48–0.86), *p* = 0.003], post-transplant maintenance [0.79 (0.68–0.92), *p* = 0.002], female gender [0.87 (0.77–0.99), *p* = 0.038], and achieving CR at best post-transplant response [0.62 (0.54–0.71), *p* < 0.001] were associated with better PFS. In contrast, lambda light chain type [1.22 (1.09–1.41), *p* = 0.001] and high-risk cytogenetics [2.02 (1.65–2.47), *p* < 0.001] were associated with worse PFS. Similarly, in multivariable analysis for OS for all patients (Table 3), post-transplant maintenance [0.83 (0.70–0.98), *p* = 0.028], female gender [0.83 (0.71–0.97), *p* = 0.020], and achieving CR at best post-transplant response [0.52 (0.44–0.61), *p* < 0.001] were associated with better OS. In contrast, lambda light chain type [1.20 (1.03–1.40), *p* = 0.022], high-risk cytogenetics [2.17 (1.72–2.73), *p* < 0.001], high burden of disease in the bone marrow [1.21 (1.03–1.43), *p* = 0.020] and HCT-CI > 3 [1.21 (1.00–1.45), *p* = 0.047] were associated with worse OS.

**Table 2:** Summary of multivariable assessment for progression free survival.

Measure	Hazard ratio (95% CI)	*p*-value
Age at autoHCT (continuous)	1.01 (1.00–1.02)	<0.001
Gender		
Male	Ref	
Female	0.87 (0.77–0.99)	0.038
Year of autoHCT		
<2010	Ref	
≥2010	0.87 (0.75–1.02)	0.09
Light chain type		
Kappa	Ref	
Lambda	1.24 (1.09–1.41)	0.001
Biclonal	0.58 (0.27–1.23)	0.16
Unknown	0.43 (0.14–1.37)	0.15
Cytogenetic risk		
Standard	Ref	
High	2.02 (1.65–2.47)	<0.001
Unknown	0.99 (0.79–1.24)	0.90
Bone marrow plasma cell burden		
<50%	Ref	
≥50%	1.15 (1.00–1.31)	0.053
Unknown	1.16 (0.89–1.51)	0.26
R-ISS		
I	Ref	
II	1.06 (0.87–1.28)	0.56
III	1.40 (0.97–2.03)	0.07
Unknown	1.44 (1.19–1.74)	<0.001
LDH		
Normal	Ref	
>ULN	1.16 (0.91–1.48)	0.22
Unknown	0.98 (0.84–1.14)	0.80
Creatinine		
≤2	Ref	
>2	0.83 (0.65–1.06)	0.13
Unknown	0.78 (0.58–1.04)	0.09
${{\boldsymbol{\beta }}}_{{\bf{2}}}$ microglobulin (continuous)	1.01 (1.00–1.03)	0.08
Induction regimen^a^		
VRD	Ref	
Non-VRD	1.09 (0.91–1.30)	0.36
Prior MRD/response		
Other^b^	Ref	
Not detected/≥VGPR	0.64 (0.48–0.86)	0.003
Maintenance^c^		
Yes vs. No	0.79 (0.68–0.92)	0.002
Hematologic best response^c^		
CR vs. non-CR	0.62 (0.54–0.71)	<0.001

*autoHCT* autologous hematopoietic stem cell transplant, *CI* confidence interval, *CR* complete response, *LDH* lactate dehydrogenase, *MRD* minimal residual disease, *Ref* reference level, *R-ISS* Revised International Staging System, *ULN* upper limit normal, *VGPR* very good partial response, *VRD* bortezomib, lenalidomide, dexamethasone.

^a^Induction regimens were reclassified into two categories: VRD vs. non-VRD.

^b^Defined as: [patients with a response of <VGPR with any MRD status prior to transplant] OR [patients with a response of ≥VGPR and MRD positive status prior to transplant].

^c^Included as a time-dependent variable in the model.

**Table 3:** Summary of multivariable assessments for overall survival.

Measure	Hazard ratio (95% CI)	*p*-value
Age at autoHCT (continuous)	1.03 (1.02–1.04)	<0.001
Gender		
Male	Ref	
Female	0.83 (0.71–0.97)	0.020
Year of autoHCT		
<2010	Ref	
≥2010	1.17 (0.96–1.41)	0.12
Light chain type		
Kappa	Ref	
Lambda	1.20 (1.03–1.40)	0.022
Biclonal	0.53 (0.20–1.43)	0.21
Unknown	0.59 (0.14–2.39)	0.46
Cytogenetic risk		
Standard	Ref	
High	2.17 (1.72–2.73)	<0.001
Unknown	1.01 (0.77–1.32)	0.94
Bone marrow plasma cell burden		
<50%	Ref	
≥50%	1.21 (1.03–1.43)	0.020
Unknown	1.13 (0.83–1.55)	0.44
R-ISS		
I	Ref	
II	1.17 (0.90–1.52)	0.23
III	1.46 (0.96–2.22)	0.08
Unknown	2.35 (1.84–3.00)	<0.001
HCT-CI score		
≤3	Ref	
>3	1.21 (1.00–1.45)	0.047
LDH		
Normal	Ref	
>ULN	1.57 (1.20–2.06)	0.001
Unknown	1.10 (0.92–1.32)	0.28
Creatinine		
≤2	Ref	
>2	0.96 (0.73–1.25)	0.74
Unknown	0.67 (0.48–0.93)	0.017
${{\boldsymbol{\beta }}}_{{\bf{2}}}$ microglobulin (continuous)	1.02 (1.00–1.04)	0.020
Induction regimen^a^		
VRD	Ref	
Non-VRD	1.20 (0.96–1.52)	0.11
Prior MRD/response		
Other^b^	Ref	
Not detected/≥VGPR	1.04 (0.72–1.48)	0.85
Maintenance^c^		
Yes vs. No	0.83 (0.70–0.98)	0.028
Hematologic best response^c^		
CR vs. non-CR	0.52 (0.44–0.61)	<0.001

*autoHCT* autologous hematopoietic stem cell transplant, *CI* confidence interval, *CR* complete response, *HCT-CI* hematopoietic cell transplant comorbidity index, *LDH* lactate dehydrogenase, *MRD* minimal residual disease, *Ref* reference level, *R-ISS* Revised International Staging System, *ULN* upper limit normal, *VGPR* very good partial response, *VRD* bortezomib, lenalidomide, dexamethasone.

^a^Induction regimens were reclassified into two categories: VRD vs. non-VRD.

^b^Defined as: [patients with a response of <VGPR with any MRD status prior to transplant] OR [patients with a response of ≥VGPR and MRD positive status prior to transplant].

^c^Included as a time-dependent variable in the model.

Univariate analyses for PFS and for OS are presented in Supplementary Tables 2 and 3.

## Discussion

Although MM is considered an incurable disease, in the present study we identified a distinct subset of patients, approximately 15% of the cohort, who had a median PFS of >14 years after induction therapy and autoHCT. These LTR were younger, more likely to have R-ISS stage I disease, standard-risk cytogenetics, lower burden of disease in the bone marrow and more often received post-transplant maintenance. LTR also had better pre- and post-transplant responses, including higher rates of MRD negativity prior to autoHCT.

Previous studies have also reported on MM patients who achieved durable remission after treatment. A comparison of the results to several key studies that evaluated LTR in MM is presented in Table 4 [4–6]. Although there is no clear criteria to define LTR, all these studies including the present one, used a PFS of at least five to eight years as a cut-off between LTR and non-LTR. Similar to our study, the report from the Mayo Clinic used a PFS of at least eight years to define LTR [6]. Despite differences across studies, the LTR population shared some common features, including younger age, lower likelihood of high-risk cytogenetic abnormalities, and lower disease stage.

**Table 4:** Selected publications on patients with multiple myeloma with long-term remission.

Study	Center	Population studied	LTR *n* (%); definition	LTR characteristic (compared to non-LTR)	Induction (in LTR)	Post-autoHCT Maintenance (in LTR)	Median PFS (in LTR)
Pasvolsky et al. (2024)	MD Anderson Cancer Center, Houston, TX, USA	1576 patients with NDMM that received upfront autoHCT, between 2000-2014	244 (15%), PFS ≥ 8 years following autoHCT	Younger age, less HRCA (4%), lower R-ISS, lower BM plasma cell infiltration	Imid + dexa (29%), VRD (18%), BOR+dexa (13%)	63%	169.3 months (95% CI 153.1-NR)
Oliver-Caldes et al. (2022)	Hospital Clinic de Barcelona, Spain	250 patients with NDMM that received upfront autoHCT, between 1990-2015	54 (21.6%); sustained response for ≥5 years following autoHCT	Younger age, female gender, better ECOG, lower ISS stage, lower BM plasma cell infiltration, lower serum calcium CRP and LDH [9% HRCA]	LEN-based (21%), BOR-based (55%)	57.4%	191 months for non-relapsers, 24 months for relapsers^a^
Paquin et al. (2020)	Mayo Clinic, Rochester, MN, USA	509 patients with NDMM that received autoHCT within 12 months, between 1998-2006	46 (9%); PFS > 8 years following autoHCT without maintenance	Median age 57 years, 61% female, 54% ISS stage I^b^ [2.5% HRCA]	Td (35%), chemotherapy (33%), high-dose dexa (22%)	0%	13.8 years (95% CI 10.5-18.5)
Terpos et al. (2020)	Alexandra General Hospital, Greece	406 patients with NDMM, between 1994-2010^c^	36 (8.8%); PFS ≥ 7 years	Younger age, better ECOG, higher Hb, better CrCl, lower ISS, less HRCA (0%), more normal pattern of marrow infiltration (on MRI)	Chemotherapy/Bor-based/Thal-based/LEN-based^d^	50%	10 years

^a^Landmark analysis starting at 5-years post-autoHCT; relapsers/non-relapsers within LTR group.

^b^Not compared to non-LTR.

^c^61% of LTR and 23% of non-LTR received upfront autoHCT.

^d^Percentages not provided.

Interestingly, even in these LTR patients in our cohort we did not see a plateau in the Kaplan–Meier curves for PFS, underscoring the fact that patients with MM continue to experience disease progression even after a long remission exceeding eight years. In addition to the disease characteristics associated with LTR as noted above, several investigators have studied the immunological factors that may contribute to a durable remission. Bryant et al. compared peripheral blood samples of 20 MM patients who survived more than 10 years from diagnosis, 50% of whom received autoHCT, to samples of other MM patients and a group of age-matched healthy controls [8]. Long-term survivors had a high frequency of cytotoxic T-cell clonal expansions and in vitro stimulation-induced proliferation, and a lower Treg/Th17 ratio. Arteche-López et al. compared peripheral blood samples of 13 MM patients who had at least 6 years of PFS following autoHCT to those of healthy blood donors [9]. Samples from LTR expressed a unique immune signature, including a higher proportion of CD4^+^ and CD8^+^ effector memory T-cells, a particular redistribution of inhibitory and activating receptors in NK-cells, an increase in naïve B cells and a reduction in marginal zone-like and class-switched memory B-cells. Another study revealed unique findings in the bone marrow of LTR, with an increase in the number B-cell precursors, plasma cells, dendritic cells and tissue macrophages, compared to patients with active MM [10]. Taken together, these findings suggest that a potential immune-based profile may contribute to a durable remission in MM.

Most patients in the present study received maintenance treatment following autoHCT. Maintenance with lenalidomide is standard of care based on the results of phase III randomized trials and a meta-analysis [11, 12]. The current practice in the United States is to continue lenalidomide maintenance indefinitely. In a recent retrospective analysis, we showed that the benefit of lenalidomide maintenance can be seen even after 5 years post-autoHCT [13]. The downside of prolonged maintenance therapy, however, include financial burden, frequent laboratory testing, lenalidomide-related adverse effects, and a significant risk of second primary malignancies [13, 14]. Based on our current understanding of LTR in MM, perhaps an LTR phenotype or favorable profile could be defined, and patients with these criteria could be preferentially enrolled in clinical trials evaluating fixed-duration or MRD-guided therapy [15, 16]. Some of these approaches could incorporate quadruplet-based induction [17, 18], chimeric antigen T (CAR-T) cells, or bispecific antibodies, which have all shown unprecedented responses in recent clinical trials [19–21]. This approach, if successful, may minimize the toxicities from indefinite therapy in this potentially favorable risk group.

With the availability of many novel and effective options, including CAR-T cells and bispecific antibodies, the role of autoHCT for MM is justifiably questioned [22]. However, our study highlights the fact that autoHCT can induce durable remission in a significant proportion of patients, with a well-documented safety profile over three decades, and low (<1%) non-relapse mortality [23]. In this study, we also showed that LTR patients had deep pre- and post-transplant responses, with a higher rate of ≥VGPR and MRD negativity prior to autoHCT, which have previously been shown to predict better post autoHCT survival outcomes [24–26]. These results support the continuing role of autoHCT in MM.

Our study has several limitations. First, being a retrospective analysis, it has inherent biases and heterogeneity in treatments. Second, with a minimum of eight-years of follow-up as an eligibility criterion, we only included patients who were transplanted up to 2014. Some of these patients received induction that may be considered suboptimal by contemporary standards, exemplified by the use of a doublet induction in 61% of patients. Similarly, 37% of patients did not receive any maintenance therapy. Furthermore, most patients had missing MRD data since MRD testing was not routinely performed during the study period.

In conclusion, we identified a distinct subset of patients, approximately 15% of the cohort, who had a long median PFS of >14 years after induction therapy and autoHCT. These LTR were younger, more likely to have R-ISS stage I disease, standard-risk cytogenetics, and more often received post-transplant maintenance.

### Supplementary information

Supplementary Table 1

Supplementary Table 2

Supplementary Table 3

Supplementary Figure 1

## Data Availability

The datasets generated during and/or analyzed during the current study are available from the corresponding author on reasonable request.
