# Supplementary material for: Multiple myeloma patients with a long remission after autologous hematopoietic stem cell transplantation
Source: Blood Cancer J. 2024 May 17;14(1):82. doi: 10.1038/s41408-024-01062-2 (PMC11101444; doi:10.1038/s41408-024-01062-2)
Supplement: Supplementary file 1 — Supplementary Table 1 [file 41408_2024_1062_MOESM1_ESM.docx]

Supplementary Table 1: Causes of death among long-term responders

| **Cause of death (Total=37)** | **Frequency n (%)** |
| --- | --- |
|  |  |
| Progression of multiple myeloma | 13 (35) |
| Second primary malignancy | 8 (22) |
| Infection | 3 (8) |
| Organ failure | 2 (5) |
| Other | 3 (8) |
| Unknown | 8 (22) |
