# Supplementary material for: Multiple myeloma patients with a long remission after autologous hematopoietic stem cell transplantation
Source: Blood Cancer J. 2024 May 17;14(1):82. doi: 10.1038/s41408-024-01062-2 (PMC11101444; doi:10.1038/s41408-024-01062-2)
Supplement: Supplementary file 2 — Supplementary Table 2 [file 41408_2024_1062_MOESM2_ESM.docx]

Supplementary Table 2: Summary of Progression Free Survival: Univariate Assessments.

| **Measure** | **Median (95% CI)** | **p-value** | **Hazard Ratio (95% CI)** | **p-value** |
| --- | --- | --- | --- | --- |
|  | **(in months)** |  |  |  |
| **PFS-All** | 33.9 (31.4 – 36.0) |  |  |  |
| **Age at autoHCT (continuous)** |  |  | 1.00 (1.00 – 1.01) | 0.09 |
| **LTR** |  |  |  |  |
| Yes | 169.3 (153.1 – NE) |  |  |  |
| No | 26.5 (25.2 – 28.1) |  |  |  |
| **Gender** |  | 0.042 |  |  |
| Male | 31.4 (28.5 – 35.0) |  | Ref |  |
| Female | 36.4 (33.1 – 39.1) |  | 0.89 (0.80 – 1.00) | 0.042 |
| **Race** |  | 0.63 |  |  |
| Black | 33.2 (26.5 – 40.3) |  | Ref |  |
| Non-Black | 33.9 (31.1 – 36.3) |  | 1.04 (0.90 – 1.20) | 0.63 |
| **Year of autoHCT** |  | <0.001 |  |  |
| <2010 | 27.6 (25.7 – 30.7) |  | Ref |  |
| ≥2010 | 41.4 (37.6 – 45.8) |  | 0.72 (0.64 – 0.80) | <0.001 |
| **Light chain type** |  | <0.001 |  |  |
| Kappa | 35.9 (32.6 – 38.5) |  | Ref |  |
| Lambda | 30.5 (26.5 – 34.2) |  | 1.23 (1.10 – 1.38) | <0.001 |
| Biclonal | 75.4 (35.7 – NE) |  | 0.56 (0.29 –1.07) | 0.08 |
| **Cytogenetic risk** |  | <0.001 |  |  |
| Standard | 37.9 (35.8 – 40.4) |  | Ref |  |
| High | 18.4 (14.4 – 23.7) |  | 1.88 (1.59 – 2.22) | <0.001 |
| **Bone marrow plasma cell burden** |  | 0.002 |  |  |
| < 50% | 36.6 (34.6 – 39.0) |  | Ref |  |
| ≥ 50% | 30.5 (26.1 – 32.6) |  | 1.20 (1.07 – 1.35) | 0.002 |
| **R-ISS** |  | <0.001 |  |  |
| I | 52.2 (42.8 – 63.3) |  | Ref |  |
| II | 38.3 (31.6 – 43.7) |  | 1.28 (1.07 – 1.52) | 0.007 |
| III | 21.6 (12.8 – 30.9) |  | 2.03 (1.50 – 2.76) | <0.001 |
| **HCT-CI score** |  | 0.31 |  |  |
| ≤3 | 33.8 (31.0 – 36.3) |  | Ref |  |
| >3 | 35.0 (27.9 – 38.6) |  | 1.07 (0.94 – 1.23) | 0.31 |
| **LDH** |  | 0.004 |  |  |
| Normal | 37.5 (34.5 – 40.0) |  | Ref |  |
| >ULN | 27.0 (21.3 – 30.8) |  | 1.35 (1.10 – 1.66) | 0.004 |
| **Creatinine** |  | 0.058 |  |  |
| ≤2 | 35.6 (32.0 – 37.5) |  | Ref |  |
| >2 | 28.3 (24.5 – 34.4) |  | 1.16 (0.99 – 1.36) | 0.059 |
| $\boldsymbol{\beta}_{\boldsymbol{2}}$ **microglobulin (continuous)** |  |  | 1.02 (1.01 – 1.03) | 0.001 |
| **Bone lesions** |  | 0.98 |  |  |
| 0 | 33.8 (28.1 – 36.6) |  | Ref |  |
| 1-3 | 33.1 (28.4 – 36.7) |  | 0.99 (0.86 – 1.13) | 0.84 |
| >3 | 33.9 (30.9 – 37.9) |  | 0.99 (0.85 – 1.14) | 0.85 |
| **Induction regimen** |  | <0.001 |  |  |
| VRD | 43.5 (37.1 – 54.7) |  | Ref |  |
| Chemo | 21.5 (18.4 – 28.1) |  | 1.95 (1.57 – 2.44) | <0.001 |
| ImiD+Dexa | 30.7 (26.5 – 33.9) |  | 1.46 (1.23 – 1.75) | <0.001 |
| VCD | 37.2 (27.2 – 48.3) |  | 1.15 (0.92 – 1.45) | 0.22 |
| Vd | 40.8 (34.6 – 46.0) |  | 1.20 (0.98 – 1.48) | 0.08 |
| VTD | 29.4 (22.6 – 36.6) |  | 1.30 (1.02 – 1.66) | 0.034 |
| Other | 30.4 (25.4 – 36.3) |  | 1.32 (1.07 – 1.64) | 0.009 |
| **Hematologic response prior to autoHCT** |  | <0.001 |  |  |
| CR/sCR | 52.0 (40.8 – 65.0) |  | Ref |  |
| nCR/VGPR | 36.6 (31.6 – 40.6) |  | 1.38 (1.09 – 1.74) | 0.007 |
| PR | 30.7 (27.5 – 33.8) |  | 1.73 (1.38 – 2.17) | <0.001 |
| SD | 26.2 (20.5 – 37.1) |  | 1.67 (1.22 – 2.28) | 0.001 |
| PD | 37.0 (5.7 – 47.1) |  | 2.55 (0.81 – 8.06) | 0.11 |
| **MRD status prior to autoHCT** |  | <0.001 |  |  |
| Negative | 75.7 (58.0 – 97.4) |  | Ref |  |
| Positive | 47.7 (39.2 – 59.5) |  | 1.55 (1.19 – 2.02) | 0.001 |
| **Prior MRD/response** |  | <0.001 |  |  |
| Other | 32.9 (30.7 – 35.7) |  | Ref |  |
| Negative/CR | NE (65.8 – NE) |  | 0.32 (0.20 – 0.51) | <0.001 |
| **Prior MRD/response** |  | < 0.001 |  |  |
| Other | 32.0 (29.7 – 34.5) |  | Ref |  |
| Negative/≥VGPR | 76.1 (56.7 – 109.1) |  | 0.46 (0.36 – 0.59) | < 0.001 |
| **Maintenance^a^** |  |  |  |  |
| Yes vs. No |  |  | 0.78 (0.70 – 0.87) | <0.001 |
| Rev with or without Dexa vs. other non-Rev maintenance |  |  | 0.73 (0.65 – 0.82) | <0.001 |
| **Hematologic response at day 100^a^** |  |  |  |  |
| CR vs. non-CR |  |  | 0.69 (0.61 – 0.78) | <0.001 |
| **Hematologic best response^a^** |  |  |  |  |
| CR vs. non-CR |  |  | 0.58 (0.52 – 0.65) | <0.001 |
| **MRD status post autoHCT^a^** |  |  |  |  |
| Negative vs. Positive |  |  | 1.20 (0.69 – 2.09) | 0.51 |
| **MRD/response^a^** |  |  |  |  |
| Negative/CR vs. Other |  |  | 0.63 (0.37 – 1.08) | 0.09 |
| Negative/≥VGPR vs. Other |  |  | 1.41 (0.80 – 2.47) | 0.23 |

**Abbreviations:** autoHCT = autologous hematopoietic stem cell transplant; Chemo=chemotherapy, CI=confidence interval, CR=complete response, Dexa=dexamethasone, HCT-CI=hematopoietic cell transplant comorbidity index, ImiD=immunomodulatory drug, LDH=lactate dehydrogenase, LTR=long-term responder, MRD=minimal residual disease, nCR =near complete response, NE=not estimated/not reached, PD=progressive disease, PFS=progression free survival, PR=partial response, Ref=reference group, Rev=lenalidomide, R-ISS=Revised International Staging System, sCR=stringent complete response, SD=stable disease, ULN=upper limit normal, VCD=bortezomib, cyclophosphamide, dexamethasone, Vd=bortezomib, dexamethasone, VGPR=very good partial response, VRD=bortezomib, lenalidomide, dexamethasone, VTD=bortezomib, thalidomide, dexamethasone.

^a^ Included as a time-dependent variable in the model.
