# Supplementary material for: Multiple myeloma patients with a long remission after autologous hematopoietic stem cell transplantation
Source: Blood Cancer J. 2024 May 17;14(1):82. doi: 10.1038/s41408-024-01062-2 (PMC11101444; doi:10.1038/s41408-024-01062-2)
Supplement: Supplementary file 3 — Supplementary Table 3 [file 41408_2024_1062_MOESM3_ESM.docx]

Supplementary Table 3: Summary of Overall Survival: Univariate Assessments.

| **Measure** | **Median (95% CI)** | **p-value** | **Hazard Ratio (95% CI)** | **p-value** |
| --- | --- | --- | --- | --- |
|  | **(in months)** |  |  |  |
| **OS-All** | 99.1 (92.9 – 104.4) |  |  |  |
| **Age at autoHCT (continuous)** |  |  | 1.02 (1.01 – 1.03) | <0.001 |
| **LTR** |  |  |  |  |
| Yes | NE (208.1 – NE) |  |  |  |
| No | 81.3 (77.5 – 85.7) |  |  |  |
| **Gender** |  | 0.009 |  |  |
| Male | 94.2 (85.9 – 101.1) |  | Ref |  |
| Female | 109.2 (97.3 – 116.5) |  | 0.84 (0.74 – 0.96) | 0.009 |
| **Race** |  | 0.10 |  |  |
| Black | 107.9 (94.9 – 124.0) |  | Ref |  |
| Non-Black | 96.9 (90.5 – 103.6) |  | 1.16 (0.97 – 1.38) | 0.10 |
| **Year of autoHCT** |  | 0.009 |  |  |
| <2010 | 89.5 (82.1 – 98.3) |  | Ref |  |
| ≥2010 | 105.6 (100.9 – 113.3) |  | 0.83 (0.72 – 0.95) | 0.009 |
| **Light chain type** |  | 0.001 |  |  |
| Kappa | 104.4 (97.4 – 113.3) |  | Ref |  |
| Lambda | 86.8 (78.8 – 97.7) |  | 1.25 (1.10 – 1.43) | <0.001 |
| Biclonal | NE (70.0 – NE) |  | 0.62 (0.28 – 1.38) | 0.24 |
| **Cytogenetic risk** |  | <0.001 |  |  |
| Standard | 109.2 (102.6 – 118.3) |  | Ref |  |
| High | 52.2 (42.2 – 70.7) |  | 2.16 (1.79 – 2.61) | <0.001 |
| **Bone marrow plasma cell burden** |  | <0.001 |  |  |
| <50% | 108.6 (99.6 – 120.1) |  | Ref |  |
| ≥50% | 88.2 (81.3 – 98.6) |  | 1.32 (1.15 – 1.51) | < 0.001 |
| **R-ISS** |  | <0.001 |  |  |
| I | 177.4 (138.3 – 198.6) |  | Ref |  |
| II | 111.5 (100.7 – 129.0) |  | 1.67 (1.31 – 2.13) | <0.001 |
| III | 70.3 (44.9 – 93.4) |  | 3.26 (2.28 – 4.65) | <0.001 |
| **HCT-CI score** |  | <0.001 |  |  |
| ≤3 | 103.9 (97.3 – 111.6) |  | Ref |  |
| >3 | 82.6 (69.6 – 92.1) |  | 1.31 (1.12 – 1.53) | <0.001 |
| **LDH** |  | <0.001 |  |  |
| Normal | 112.9 (102.3 – 123.3) |  | Ref |  |
| >ULN | 70.3 (61.1 – 87.5) |  | 1.70 (1.35 – 2.14) | <0.001 |
| **Creatinine** |  | <0.001 |  |  |
| ≤2 | 103.6 (97.3 – 109.8) |  | Ref |  |
| >2 | 74.5 (63.3 – 91.3) |  | 1.43 (1.20 – 1.71) | <0.001 |
| $\boldsymbol{\beta}_{\boldsymbol{2}}$ **microglobulin (continuous)** |  |  | 1.03 (1.02 – 1.05) | <0.001 |
| **Bone lesions** |  | 0.41 |  |  |
| 0 | 102.6 (94.2 – 120.7) |  | Ref |  |
| 1-3 | 99.4 (88.7 – 109.2) |  | 1.09 (0.92 – 1.28) | 0.33 |
| >3 | 95.6 (84.2 – 105.8) |  | 1.12 (0.95 – 1.33) | 0.19 |
| **Induction regimen** |  | <0.001 |  |  |
| VRD | 122.7 (109.3 – 168.3) |  | Ref |  |
| Chemo | 73.1 (56.0 – 86.9) |  | 2.01 (1.54 – 2.61) | <0.001 |
| ImiD+Dexa | 100.5 (84.6 – 111.6) |  | 1.29 (1.03 – 1.61) | 0.024 |
| VCD | 101.1 (83.2 – 126.8) |  | 1.33 (1.00 – 1.77) | 0.048 |
| Vd | 98.6 (88.0 – 109.8) |  | 1.30 (1.01 – 1.69) | 0.044 |
| VTD | 96.5 (77.2 – 115.7) |  | 1.37 (1.03 – 1.84) | 0.031 |
| Other | 93.7 (81.2 – 110.8) |  | 1.40 (1.08 – 1.82) | 0.011 |
| **Hematologic response prior to autoHCT** |  | <0.001 |  |  |
| CR/sCR | 167.4 (108.4 – NE) |  | Ref |  |
| nCR/VGPR | 102.3 (92.7 – 113.0) |  | 1.48 (1.11 – 1.98) | 0.008 |
| PR | 93.4 (84.8 – 101.0) |  | 1.68 (1.27 – 2.22) | <0.001 |
| SD | 85.9 (63.8 – 115.0) |  | 1.73 (1.20 – 2.50) | 0.003 |
| PD | 40.4 (17.7 – 77.2) |  | 5.98 (1.87 – 19.14) | 0.003 |
| **MRD status prior to autoHCT** |  | 0.46 |  |  |
| Negative | 193.3 (105.6 – 193.3) |  | Ref |  |
| Positive | 130.8 (109.3 – 141.1) |  | 1.14 (0.80 – 1.62) | 0.46 |
| **Prior MRD/response** |  | 0.006 |  |  |
| Other | 97.9 (91.8 – 103.8) |  | Ref |  |
| Not detected/CR | NE (97.7 – NE) |  | 0.44 (0.24 – 0.80) | 0.007 |
| **Prior MRD/response** |  | 0.001 |  |  |
| Other | 96.5 (90.3 – 102.6) |  | Ref |  |
| Not detected/≥VGPR | 115.9 (105.6 – 193.3) |  | 0.60 (0.44 – 0.82) | 0.001 |
| **Maintenance Group^a^** |  |  |  |  |
| Yes vs. No |  |  | 0.83 (0.73 – 0.95) | 0.006 |
| Rev with or without Dexa vs. other non-Rev maintenance |  |  | 0.78 (0.67 – 0.90) | <0.001 |
| **Hematologic response at day 100^a^** |  |  |  |  |
| CR vs. non-CR |  |  | 0.61 (0.52 – 0.71) | <0.001 |
| **Hematologic best response^a^** |  |  |  |  |
| CR vs. non-CR |  |  | 0.51 (0.44 – 0.58) | <0.001 |
| **MRD status post autoHCT^a^** |  |  |  |  |
| Negative vs. Positive |  |  | 1.12 (0.52 – 2.45) | 0.77 |
| **MRD/response^a^** |  |  |  |  |
| Negative/CR vs. Other |  |  | 0.74 (0.37 – 1.50) | 0.41 |
| Negative/≥VGPR vs. Other |  |  | 1.18 (0.54 – 2.57) | 0.69 |

**Abbreviations:** autoHCT=autologous hematopoietic stem cell transplant; Chemo=chemotherapy, CI=confidence interval, CR=complete response, Dexa=dexamethasone, HCT-CI=hematopoietic cell transplant comorbidity index, ImiD=immunomodulatory drug, LDH=lactate dehydrogenase, LTR=long-term responder, MRD=minimal residual disease, nCR =near complete response, NE=not estimated/not reached, OS=overall survival, PD=progressive disease, PR=partial response, Ref=reference group, Rev=lenalidomide, R-ISS=Revised International Staging System, sCR=stringent complete response, SD=stable disease, ULN=upper limit normal, VCD=bortezomib, cyclophosphamide, dexamethasone, Vd=bortezomib, dexamethasone, VGPR=Very good partial response, VRD=bortezomib, lenalidomide, dexamethasone, VTD=bortezomib, thalidomide, dexamethasone.

^a^ Included as a time-dependent variable in the model.
