# Supplementary figures and images for: Multiple myeloma patients with a long remission after autologous hematopoietic stem cell transplantation
Source: Blood Cancer J. 2024 May 17;14(1):82. doi: 10.1038/s41408-024-01062-2 (PMC11101444; doi:10.1038/s41408-024-01062-2)

## Slide 1
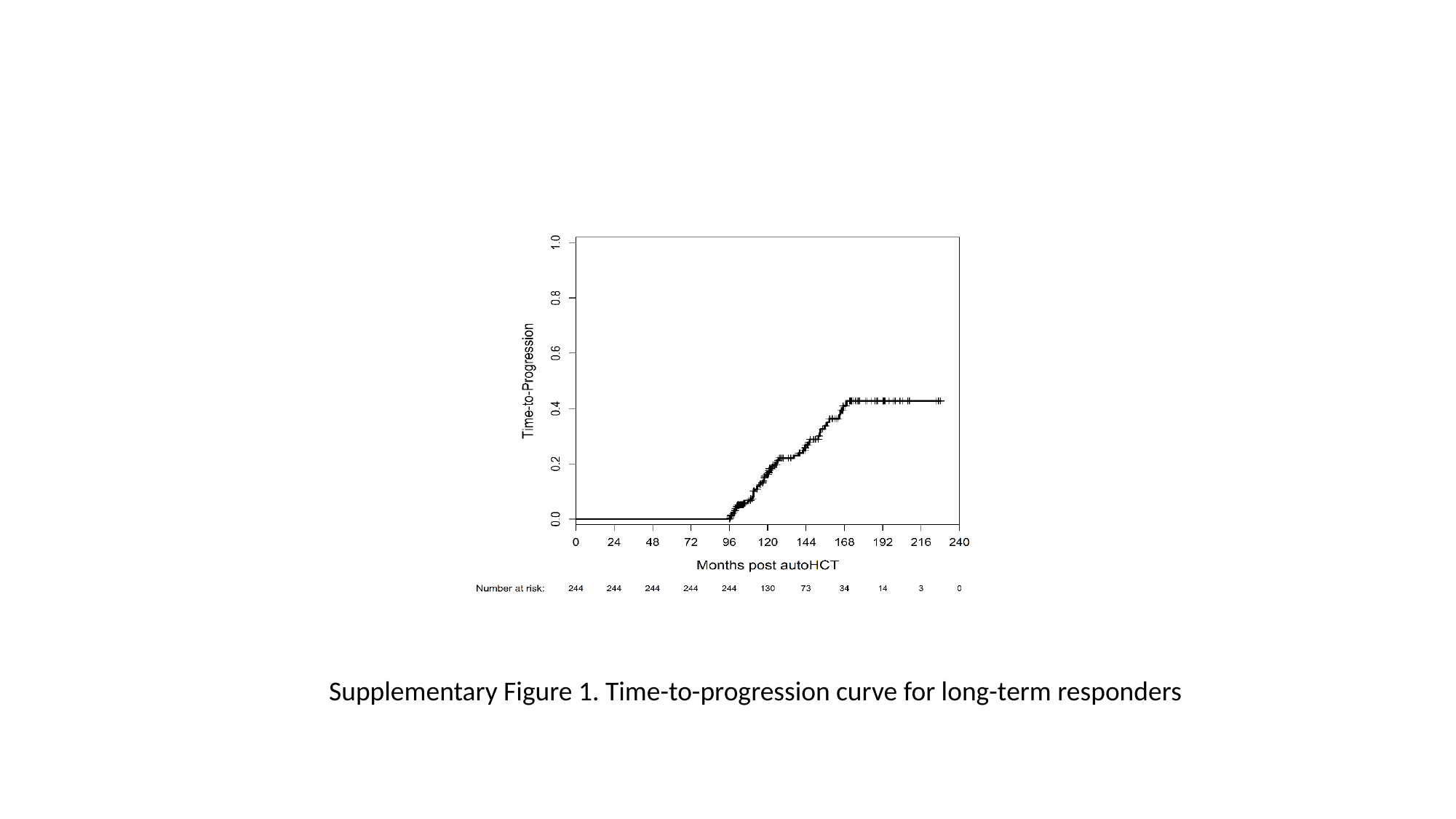

Supplementary Figure 1. Time-to-progression curve for long-term responders

Supplement: Supplementary file 4 — Supplementary Figure 1 [file 41408_2024_1062_MOESM4_ESM.pptx]
